# Potassium 4-azidobenzene­sulfonate^1^

**DOI:** 10.1107/S1600536814015669

**Published:** 2014-07-11

**Authors:** Frank Biesemeier, Gertraud Geiseler, Klaus Harms, Ulrich Müller

**Affiliations:** a Fachbereich Chemie, Philipps-Universität Marburg, 35032 Marburg, Germany

**Keywords:** crystal structure

## Abstract

In, K^+^·SO_3_–*p*-C_6_H_4_–N_3_^−^, the conformation angle of the azido group with respect to the benzene ring is 19.1 (3)°, so that the anion is chiral within the crystal structure. In addition, the crystal structure is also chiral (Sohncke space group). The potassium ion is coordinated by three closer O atoms from three different sulfonyl groups [K⋯O 2.6486 (17) to 2.7787 (17) Å], three more distant O atoms [K⋯O 2.959 (2) to 3.206 (2) Å] and three N atoms at 3.073 (2) to 3.268 (2) Å. The anions are packed into layers perpendicular to *b*, only O and N atoms being at the surface of the layers. The K^+^ ions are located between the layers.

## Related literature   

For the synthesis, see: Biesemeier *et al.* (2003 ▶). For the crystal structures of the same anion with different cations, see: Biesemeier *et al.* (2004*a* ▶,*b* ▶,*c* ▶).
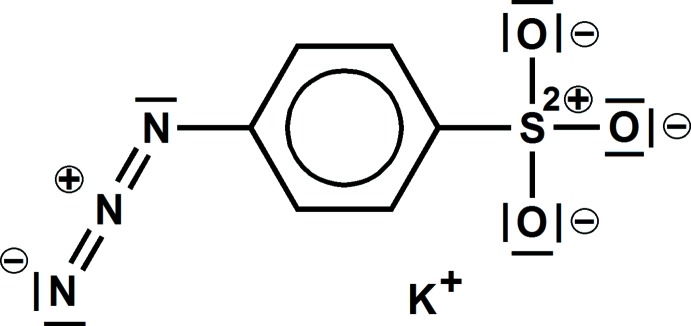


## Experimental   

### 

#### Crystal data  

K(C_6_H_4_N_3_O_3_S)
*M* *_r_* = 237.28
Orthorhombic, 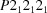

*a* = 5.4220 (5) Å
*b* = 7.9937 (7) Å
*c* = 19.267 (2) Å
*V* = 835.08 (15) Å^3^
*Z* = 4
Mo *K*α radiation
μ = 0.87 mm^−1^
*T* = 193 K
0.55 × 0.33 × 0.25 mm

#### Data collection  

STOE IPDS2 diffractometer
Absorption correction: integration (*XPREP*; Bruker, 2012 ▶) *T*_min_ = 0.740, *T*_max_ = 0.841
6140 measured reflections
1639 independent reflections
1591 reflections with *I* > 2σ(*I*)
*R*_int_ = 0.038

#### Refinement  

*R*[*F* ^2^ > 2σ(*F* ^2^)] = 0.018
*wR*(*F* ^2^) = 0.048
*S* = 1.09
1639 reflections
143 parameters
All H-atom parameters refined
Δρ_max_ = 0.32 e Å^−3^
Δρ_min_ = −0.25 e Å^−3^
Absolute structure: Flack *x* determined using 636 quotients [(*I* ^+^)−(*I* ^−^)]/[(*I* ^+^)+(*I* ^−^)] (Parsons & Flack, 2004 ▶)
Absolute structure parameter: 0.004 (16)

### 

Data collection: *X-AREA* (Stoe & Cie, 2005 ▶); cell refinement: *X-AREA*; data reduction: *X-AREA*; program(s) used to solve structure: *SHELXS97* (Sheldrick, 2008 ▶); program(s) used to refine structure: *SHELXL2013* (Sheldrick, 2008 ▶); molecular graphics: *DIAMOND* (Brandenburg, 2000 ▶); software used to prepare material for publication: *SHELXL2013*.

## Supplementary Material

Crystal structure: contains datablock(s) global, I. DOI: 10.1107/S1600536814015669/hp2068sup1.cif

Structure factors: contains datablock(s) I. DOI: 10.1107/S1600536814015669/hp2068Isup2.hkl

CCDC reference: 1012203

Additional supporting information:  crystallographic information; 3D view; checkCIF report

## Figures and Tables

**Fig. 1.**
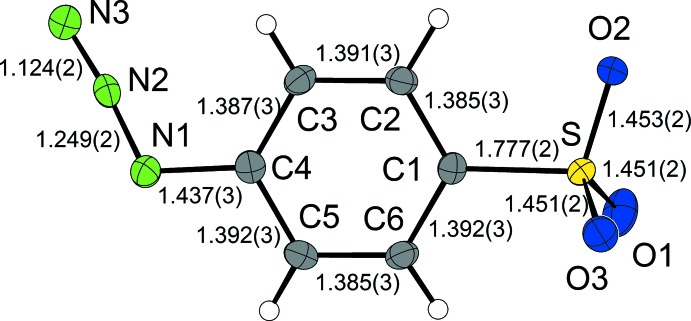
Die Molekularstruktur des [O3S–C6H4–N3]–-Ions. Schwingungsellipsoide mit 50% Aufenthaltswahrscheinlichkeit bei 193 K. Bindungslängen in Å.

**Fig. 2.**
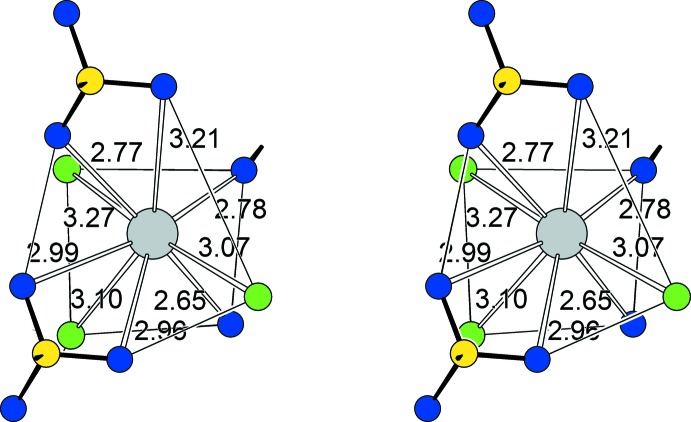
Das Koordinationspolyeder um ein K+-Ion bis 3,30 Å. Kontaktabstände in Å. Man betrachte das Stereobild über Kreuz (linkes Bild für das rechte Auge).

**Fig. 3.**
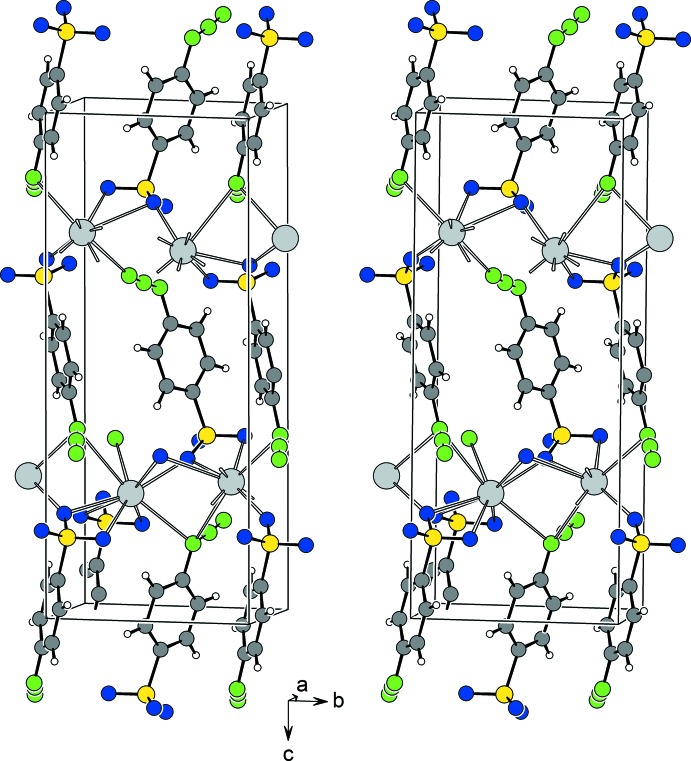
Die Kristallpackung in K[O3S–C6H4–N3]. Beim K+-Ion im der Mitte unten sind alle koordinierten Atome bis zu einem Abstand von 3,30 Å eingezeichnet. Stereobild über Kreuz betrachten.

## supplementary crystallographic information

### S1.1. Synthese und Kristallisation

Zur Synthese siehe Biesemeier *et al.* (2003). Beim letzten Schritt, der Kristallisation von K[O_3_S–C_6_H_4_–N_3_] aus Methanol, wurden die Kristalle aber nicht durch Abkühlen der Lösung erhalten, sondern durch partielle Verdunstung des Methanols bei Zimmertemperatur. Nach ihren Röntgendiagrammen haben die erhaltenen Kristalle die gleiche Struktur bei Zimmertemperatur und bei 193 K.

Elementaranalyse: C 30,30 % (ber. 30,37 %), H 1,92 % (ber. 1,70 %), N 17,68 % (ber. 17,71 %).

Infrarot-Spektrum (KBr-Pressling und Nujol-Verreibung, Absorptionsmaxima in cm^–1^): 2142 (ν_as_ N_3_), 1591, 1493, 1272 (ν_s_ N_3_), 1207 (ν_as_ SO_3_), 1143, 1117 (ν_s_ SO_3_), 1041, 1005, 831, 729, 711 (ν CS), 650 (δ N_3_), 569 (δ SO_3_), 524.

### S1.2. Verfeinerung

Die Kristalldaten und Angaben zur Messung und Strukturverfeinerung sind in Tabelle 1 zusammengestellt.

### S2. Ergebnisse und Diskussion

Die Titelverbindung wurde neben Kalium-4-penta­zolylbenzolsulfonat (K^+^[O_3_S–C_6_H_4_–N_5_]^–^) wie beschrieben hergestellt (Biesemeier *et al.*, 2003), aus 4-Diazo­niumbenzolsulfonat und Natriumazid und anschließender Fällung mit KOH aus methano­lischer Lösung bei –50°C. Das [O_3_S–C_6_H_4_N_3_]^–^ -Ion eignet sich um K^+^ von Na^+^ durch Fällungsreaktion aus methano­lischer Lösung zu trennen. Durch Extraktion mit Aceton, Eindampfen der Lösung und Umkristallisation aus Methanol wurden Kristalle der Titelverbindung erhalten. Wird das Umkristallisieren durch Abkühlen der methano­lischen Lösung auf –45°C bewirkt, so entstehen Kristalle, die Satellitenreflexe zeigen. Lässt man dagegen das Methanol bei Zimmertemperatur verdunsten, treten keine Satellitenreflexe auf.

Das [O_3_S–C_6_H_4_–N_3_]^–^ -Ion hat dieselbe Struktur, wie sie schon bei [THF-K-18-Krone-8][O_3_S–C_6_H_4_–N_3_] (Biesemeier *et al.*, 2003) und mit weiteren Kationen (Biesemeier *et al.*, 2004*a*,*b*,*c*) gefunden wurde (Fig. 1). Die Azido­gruppe ist leicht geknickt (Winkel N–N–N 174,3°). Der Konformationswinkel der Azido­gruppe relativ zum aromatischen Ring beträgt 19,1°. Damit ist das Molekül im Kristall chiral; im Kristall liegt kein Racemat vor (Sohncke-Raumgruppe P2_1_2_1_2_1_).

Ein Kalium-Ion ist von drei nähergelegenen O-Atomen aus drei verschiedenen Sulfonyl­gruppen koordiniert (S···O-Abstände 2,65 bis 2,78 Å). Dazu kommen drei etwas längere S···O-Kontakte zu drei weiteren O-Atomen. Von den insgesamt vier koordinierten Sulfonyl­gruppen sind zwei chelatartig an das K^+^ -Ion koordiniert. Etwas entfernter befinden sich Stickstoff-Atome (3,07, 3,10, 3,27 Å und weitere). Rechnet man nur die drei nächsten N-Atome zur Koordinationssphäre, so ist das Koordinationspolyeder ein 4-5-Polyeder, d.h. es hat ein verzerrtes Quadrat und ein gegenüberliegendes verzerrtes Fünfeck als Deckflächen (Fig. 2). Im Gegensatz dazu haben die N-Atome des [O_3_S–C_6_H_4_–N_3_]^–^ -Ions im Na[O_3_S–C_6_H_4_–N_3_] keine Kontakte mit einem Na^+^ -Ion (Biesemeier *et al.*, 2004*a*,*b*,*c*, Seite 37), ebensowenig wie mit den stark abgeschirmten Kalium-Atomen im [K-18-Krone-6-THF][O_3_S–C_6_H_4_–N_3_] (Biesemeier *et al.*, 2003).

Die [O_3_S–C_6_H_4_–N_3_]^–^ -Ionen sind zu Schichten senkrecht zur *b*-Achse gepackt. Auf den Außenseiten der Schichten befinden sich nur N- und O-Atome. Die K^+^ -Ionen befinden sich zwischen den Schichten (Fig. 3).

### Crystal data

**Table:**

K(C_6_H_4_N_3_O_3_S)	*D*_x_ = 1.887 Mg m^−^^3^
*M**_r_* = 237.28	Mo *K*α radiation, λ = 0.71069 Å
Orthorhombic, *P*2_1_2_1_2_1_	Cell parameters from 10459 reflections
*a* = 5.4220 (5) Å	θ = 2.1–26.1°
*b* = 7.9937 (7) Å	µ = 0.87 mm^−^^1^
*c* = 19.267 (2) Å	*T* = 193 K
*V* = 835.08 (15) Å^3^	Prism, yellow
*Z* = 4	0.55 × 0.33 × 0.25 mm
*F*(000) = 480	

### Data collection

**Table:**

STOE IPDS2 diffractometer	1639 independent reflections
Radiation source: sealed X-ray tube	1591 reflections with *I* > 2σ(*I*)
Graphite monochromator	*R*_int_ = 0.038
ω scans	θ_max_ = 26.1°, θ_min_ = 2.1°
Absorption correction: integration (*XPREP*; Bruker, 2012)	*h* = −6→6
*T*_min_ = 0.740, *T*_max_ = 0.841	*k* = −9→9
6140 measured reflections	*l* = −23→23

### Refinement

**Table:**

Refinement on *F*^2^	Secondary atom site location: difference Fourier map
Least-squares matrix: full	Hydrogen site location: difference Fourier map
*R*[*F*^2^ > 2σ(*F*^2^)] = 0.018	All H-atom parameters refined
*wR*(*F*^2^) = 0.048	*w* = 1/[σ^2^(*F*_o_^2^) + (0.0214*P*)^2^ + 0.2034*P*] where *P* = (*F*_o_^2^ + 2*F*_c_^2^)/3
*S* = 1.09	(Δ/σ)_max_ < 0.001
1639 reflections	Δρ_max_ = 0.32 e Å^−^^3^
143 parameters	Δρ_min_ = −0.25 e Å^−^^3^
0 restraints	Absolute structure: Flack x determined using 636 quotients [(*I*^+^)-(*I*^-^)]/[(*I*^+^)+(*I*^-^)] (Parsons & Flack, 2004)
Primary atom site location: structure-invariant direct methods	Absolute structure parameter: 0.004 (16)

### Special details

**Table:**

Geometry. All e.s.d.'s (except the e.s.d. in the dihedral angle between two l.s. planes) are estimated using the full covariance matrix. The cell e.s.d.'s are taken into account individually in the estimation of e.s.d.'s in distances, angles and torsion angles; correlations between e.s.d.'s in cell parameters are only used when they are defined by crystal symmetry. An approximate (isotropic) treatment of cell e.s.d.'s is used for estimating e.s.d.'s involving l.s. planes.

### Fractional atomic coordinates and isotropic or equivalent isotropic displacement parameters (Å^2^)

**Table:**

	*x*	*y*	*z*	*U*_iso_*/*U*_eq_	
K	−0.01018 (9)	0.67980 (6)	0.26487 (2)	0.02287 (13)	
S	0.49304 (10)	−0.10392 (6)	0.33927 (2)	0.01569 (12)	
O1	0.5157 (4)	−0.28483 (18)	0.33948 (8)	0.0325 (4)	
O2	0.2819 (3)	−0.0487 (2)	0.29887 (7)	0.0216 (3)	
O3	0.7217 (3)	−0.0217 (2)	0.32010 (8)	0.0274 (4)	
N1	0.3166 (3)	0.0942 (2)	0.63445 (9)	0.0211 (4)	
N2	0.1000 (3)	0.1318 (2)	0.65003 (9)	0.0186 (4)	
N3	−0.0882 (3)	0.1672 (3)	0.66954 (10)	0.0253 (4)	
C1	0.4320 (4)	−0.0448 (3)	0.42651 (10)	0.0170 (4)	
C2	0.2204 (4)	0.0433 (3)	0.44334 (10)	0.0193 (4)	
C3	0.1787 (4)	0.0909 (3)	0.51178 (10)	0.0195 (4)	
C4	0.3477 (4)	0.0485 (3)	0.56286 (10)	0.0179 (4)	
C5	0.5598 (4)	−0.0410 (3)	0.54628 (11)	0.0203 (4)	
C6	0.6009 (4)	−0.0873 (3)	0.47797 (11)	0.0196 (4)	
H2	0.095 (4)	0.065 (3)	0.4069 (12)	0.020 (6)*	
H3	0.047 (5)	0.153 (3)	0.5228 (12)	0.019 (6)*	
H5	0.665 (5)	−0.074 (3)	0.5797 (13)	0.021 (6)*	
H6	0.730 (5)	−0.150 (3)	0.4652 (13)	0.023 (6)*	

### Atomic displacement parameters (Å^2^)

**Table:**

	*U*^11^	*U*^22^	*U*^33^	*U*^12^	*U*^13^	*U*^23^
K	0.0214 (2)	0.0205 (2)	0.0268 (2)	−0.0031 (2)	0.0020 (2)	−0.00356 (16)
S	0.0137 (2)	0.0174 (2)	0.0159 (2)	0.0008 (2)	0.0016 (2)	−0.00066 (16)
O1	0.0519 (10)	0.0183 (8)	0.0275 (8)	0.0083 (8)	0.0062 (10)	−0.0005 (6)
O2	0.0165 (7)	0.0300 (8)	0.0183 (7)	0.0033 (7)	−0.0023 (6)	−0.0031 (6)
O3	0.0159 (7)	0.0446 (10)	0.0217 (7)	−0.0074 (7)	0.0043 (6)	−0.0003 (7)
N1	0.0184 (8)	0.0264 (9)	0.0185 (8)	−0.0001 (8)	0.0012 (7)	−0.0022 (8)
N2	0.0228 (9)	0.0187 (9)	0.0144 (8)	−0.0008 (7)	−0.0018 (7)	−0.0022 (6)
N3	0.0252 (10)	0.0290 (10)	0.0218 (9)	0.0045 (8)	0.0000 (7)	−0.0027 (8)
C1	0.0177 (10)	0.0166 (9)	0.0167 (8)	−0.0022 (8)	0.0020 (7)	−0.0003 (7)
C2	0.0162 (10)	0.0234 (10)	0.0182 (10)	0.0014 (9)	−0.0010 (7)	0.0024 (8)
C3	0.0165 (9)	0.0213 (10)	0.0208 (9)	0.0024 (9)	0.0032 (8)	−0.0001 (8)
C4	0.0178 (10)	0.0181 (10)	0.0177 (9)	−0.0044 (8)	0.0014 (7)	0.0002 (8)
C5	0.0173 (10)	0.0228 (10)	0.0207 (9)	0.0002 (8)	−0.0035 (7)	0.0014 (8)
C6	0.0147 (9)	0.0214 (11)	0.0228 (10)	0.0020 (9)	0.0012 (8)	−0.0011 (8)

### Geometric parameters (Å, º)

**Table:**

K—O1^i^	3.206 (2)	N1—C4	1.437 (3)
K—O1^ii^	2.959 (2)	N1—N2	1.249 (2)
K—O2^iii^	2.6486 (17)	N2—N3	1.124 (3)
K—O2^i^	2.7652 (18)	C1—C2	1.385 (3)
K—O3^iv^	2.7787 (17)	C1—C6	1.392 (3)
K—O3^ii^	2.9900 (19)	C2—C3	1.391 (3)
K—N1^v^	3.0731 (19)	C3—C4	1.387 (3)
K—N3^vi^	3.1001 (19)	C4—C5	1.391 (3)
K—N1^vii^	3.2680 (18)	C5—C6	1.385 (3)
S—O1	1.4514 (15)	C2—H2	0.99 (2)
S—O2	1.4530 (15)	C3—H3	0.90 (3)
S—O3	1.4510 (16)	C5—H5	0.90 (3)
S—C1	1.7772 (19)	C6—H6	0.90 (3)
			
O2^iii^—K—O2^i^	165.85 (3)	N3^vi^—K—N1^vii^	63.38 (5)
O2^iii^—K—O3^iv^	68.91 (5)	O1^i^—K—N1^vii^	90.59 (4)
O2^i^—K—O3^iv^	105.79 (5)	O3—S—O1	112.31 (12)
O2^iii^—K—O1^ii^	78.93 (5)	O3—S—O2	113.54 (9)
O2^i^—K—O1^ii^	107.90 (5)	O1—S—O2	111.77 (11)
O3^iv^—K—O1^ii^	146.17 (5)	O1—S—C1	106.14 (9)
O2^iii^—K—O3^ii^	116.42 (5)	O2—S—C1	106.21 (9)
O2^i^—K—O3^ii^	64.39 (4)	O3—S—C1	106.23 (9)
O3^iv^—K—O3^ii^	161.09 (5)	S—O1—K^viii^	99.64 (10)
O1^ii^—K—O3^ii^	47.81 (4)	S—O1—K^ix^	90.64 (9)
O2^iii^—K—N1^v^	68.35 (5)	S—O2—K^x^	153.64 (10)
O2^i^—K—N1^v^	125.79 (5)	S—O2—K^ix^	109.87 (9)
O3^iv^—K—N1^v^	97.52 (5)	S—O3—K^xi^	153.32 (10)
O1^ii^—K—N1^v^	59.72 (5)	S—O3—K^viii^	98.31 (9)
O3^ii^—K—N1^v^	101.27 (5)	N2—N1—C4	113.74 (17)
O2^iii^—K—N3^vi^	66.84 (5)	N1—N2—N3	174.3 (2)
O2^i^—K—N3^vi^	103.47 (5)	C2—C1—C6	120.18 (18)
O3^iv^—K—N3^vi^	105.97 (5)	C2—C1—S	120.73 (15)
O1^ii^—K—N3^vi^	68.90 (5)	C6—C1—S	119.09 (16)
O3^ii^—K—N3^vi^	63.54 (5)	C1—C2—C3	119.72 (19)
N1^v^—K—N3^vi^	116.14 (5)	C1—C2—H2	119.5 (14)
O2^iii^—K—O1^i^	139.77 (5)	C3—C2—H2	120.7 (14)
O2^i^—K—O1^i^	46.78 (4)	C4—C3—C2	119.89 (19)
O3^iv^—K—O1^i^	79.32 (5)	C4—C3—H3	119.6 (15)
O1^ii^—K—O1^i^	123.12 (5)	C2—C3—H3	120.5 (15)
O3^ii^—K—O1^i^	101.68 (4)	C3—C4—C5	120.59 (19)
N1^v^—K—O1^i^	92.96 (5)	C3—C4—N1	122.77 (19)
N3^vi^—K—O1^i^	148.83 (5)	C5—C4—N1	116.64 (18)
O2^iii^—K—N1^vii^	101.73 (5)	C6—C5—C4	119.3 (2)
O2^i^—K—N1^vii^	64.18 (4)	C6—C5—H5	119.9 (16)
O3^iv^—K—N1^vii^	71.73 (5)	C4—C5—H5	120.8 (16)
O1^ii^—K—N1^vii^	126.86 (5)	C5—C6—C1	120.4 (2)
O3^ii^—K—N1^vii^	89.35 (5)	C5—C6—H6	122.5 (16)
N1^v^—K—N1^vii^	167.85 (5)	C1—C6—H6	117.0 (16)
			
N2—N1—C4—C3	19.1 (3)	O1—S—C1—C2	−121.7 (2)
N2—N1—C4—C5	−161.41 (19)	O2—S—C1—C2	−2.6 (2)
C3—C4—C5—C6	0.1 (3)	O1—S—C1—C6	58.7 (2)
N1—C4—C5—C6	−179.5 (2)	O2—S—C1—C6	177.77 (17)
C5—C4—C3—C2	0.3 (3)	O3—S—C1—C2	118.55 (19)
N1—C4—C3—C2	179.8 (2)	O3—S—C1—C6	−61.1 (2)
C1—C2—C3—C4	−0.7 (3)	C4—C5—C6—C1	0.0 (3)
C3—C2—C1—C6	0.8 (3)	C2—C1—C6—C5	−0.5 (3)
C3—C2—C1—S	−178.80 (17)	S—C1—C6—C5	179.14 (18)

Symmetry codes: (i) *x*, *y*+1, *z*; (ii) *x*−1, *y*+1, *z*; (iii) −*x*, *y*+1/2, −*z*+1/2; (iv) −*x*+1, *y*+1/2, −*z*+1/2; (v) *x*−1/2, −*y*+1/2, −*z*+1; (vi) −*x*−1/2, −*y*+1, *z*−1/2; (vii) −*x*+1/2, −*y*+1, *z*−1/2; (viii) *x*+1, *y*−1, *z*; (ix) *x*, *y*−1, *z*; (x) −*x*, *y*−1/2, −*z*+1/2; (xi) −*x*+1, *y*−1/2, −*z*+1/2.
